# Effect of Knowledge/Practice of COVID-19 Prevention Measures on Return-to-Work Concerns; Attitudes About the Efficacy of Traditional Chinese Medicine: Survey on Supermarket Staff in Huanggang, China

**DOI:** 10.3389/fpubh.2021.722604

**Published:** 2021-09-16

**Authors:** Lingru Li, Yue Meng, Ji Wang, Ying Zhang, Yong Zeng, Huiqun Xiao, Jiangming He, Zhenquan Liu, Shujuan Hou, Tianxing Li, Jingbo Qin, Yini Fang, Wenqian Guo, Li'an Liu, Hui Luo, Yingshuai Li, Yanfei Zheng, Qi Wang

**Affiliations:** ^1^National Institute of Traditional Chinese Medicine Constitution and Preventive Treatment of Diseases, Beijing University of Chinese Medicine, Beijing, China; ^2^College of Chinese Medicine, Beijing University of Chinese Medicine, Beijing, China; ^3^Health Committee of Huanggang, Huanggang, China; ^4^Huangzhou Maternity and Child Health Care Hospital, Huanggang, China; ^5^Public Health Department, Huangzhou General Hospital of Huanggang, Huanggang, China; ^6^College of Chinese Classics, Beijing University of Chinese Medicine, Beijing, China; ^7^Institute for Tibetan Medicine, China Tibetology Research Center, Beijing, China

**Keywords:** COVID-19, traditional Chinese medicine, prevention, resuming work, knowledge and practice, concern, Hubei

## Abstract

**Objective:** The objective of this study was to investigate how knowledge and practice of coronavirus disease 2019 (COVID-19) prevention measures affected concerns about returning to work among supermarket staff. Attitudes about the ability of traditional Chinese medicine (TCM) to prevent COVID-19 were also assessed.

**Methods:** A cross-sectional study was conducted in Huanggang, Hubei Province, China from April 23 to 25, 2020. Participants were invited to fill out an electronic questionnaire on their cell phones.

**Results:** The results showed that from 2,309 valid questionnaires, 61.5% of participants were concerned about resuming work. Major concerns included asymptomatic infection (85.01%) and employees gathering in the workplace (78.96%). Multivariate logistic regression indicated that the female gender, having school-aged children and pregnancy were risk factors for being concerned about resuming work, while good knowledge and practice of preventive measures were protective factors. Knowledge and practice of preventive measures were positively correlated. Among preventive measures, the highest percentage of participants knew about wearing masks and washing hands. Meanwhile, 65.8% of participants expressed confidence in the ability of TCM to prevent COVID-19, where 74 and 51.3% thought there was a need and a strong need, respectively, for preventive TCM-based products. Among them, 71.5% preferred oral granules. Regarding TCM as a COVID-19 preventative, most were interested in information about safety and efficacy.

**Conclusion:** These findings suggested that promoting knowledge and practices regarding COVID-19 prevention can help alleviate concerns about returning to work. Meanwhile, TCM can feasibly be accepted to diversify COVID-19 prevention methods.

**Clinical Trial Registration:**http://www.chictr.org.cn/, identifier: ChiCTR2000031955.

## Introduction

Coronavirus disease 2019 has resulted in 171 million confirmed cases and over 3 million deaths worldwide (1). Many regions and countries adopted the policy of work stoppage and city lockdown, and they were faced with the problem of resuming work and production after the epidemic has been controlled. In China, Hubei Province was the most severely affected area at the beginning of the outbreak, leading to a regional lockdown and a large-scale shutdown.

Supermarket staff had to keep working on-site and were inevitably exposed to the flow of people because of the nature of their job. Supermarkets cannot fully shut down and can only reduce the attendance rate during the worst of the epidemic. Their staff were the first to fully return to work when the epidemic was under control. This study focused on supermarket staff in high-risk areas since coronavirus disease 2019 (COVID-19) cases in supermarkets have been reported to form disease clusters (2–5). Therefore, it is important for supermarket staff to know and practice COVID-19 prevention measures. After the outbreak was brought under control, society shifted toward resuming work and production. With increased economic activity, the number of customers increased, and supermarket staff accordingly faced an increased risk of infection. In this context, we investigated the COVID-19 prevention knowledge and practices of supermarket staff, and the factors affecting their concerns about returning to work.

In order to better ensure the resumption of work and production, the prevention of COVID-19 cannot be relaxed. At the time of this study, the vaccine had not been applied. Aside from conventional sterilization and mask protection, traditional Chinese medicine (TCM) is one of the most distinctive and promising prevention methods in China. Therefore, we also investigated attitudes toward preventing COVID-19 by means of TCM, the demand for related TCM products, and the feasibility of promoting TCM as an additional COVID-19 prevention measure.

## Methods

### Background and Participants

**Figure 1:**
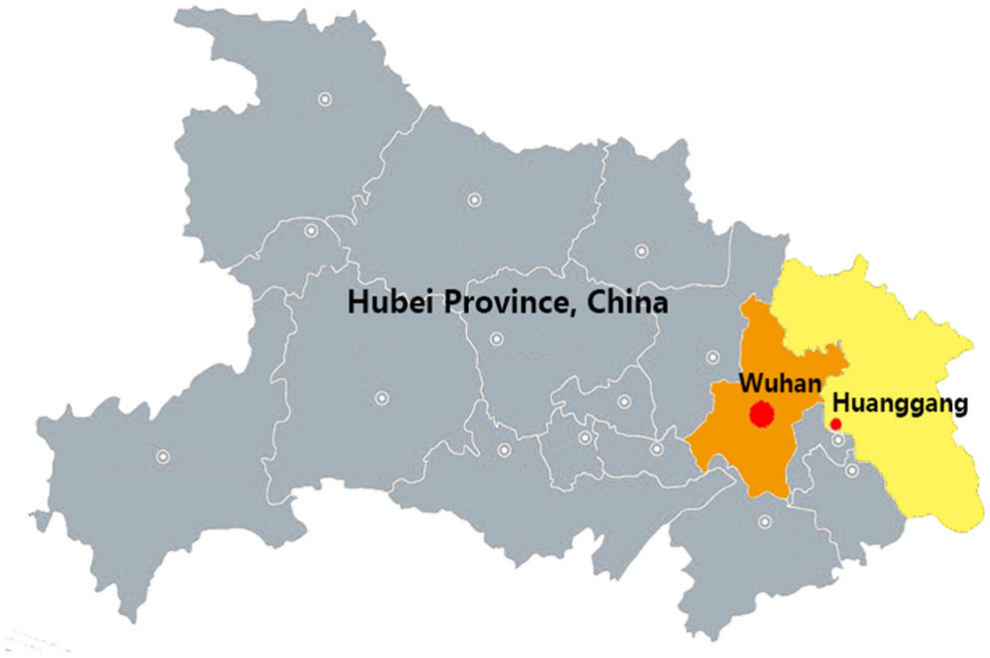
Geographical location of Huanggang, where the respondents were located.

A cross-sectional survey was conducted in Huanggang, Hubei Province, China from April 23 to 25, 2020. Huanggang is a prefecture-level city in Hubei Province, adjacent to the provincial capital Wuhan and is an important part of the Wuhan urban agglomeration (Figure 1). With a population of 7.5 million, Huanggang is the second most populous city in Hubei Province after Wuhan and one of the cities hardest hit by COVID-19 (6). The city had a total of 2,907 confirmed COVID-19 cases and 125 deaths. On March 18, the last three COVID-19 patients in Huanggang were discharged from the hospital (7). However, the government began to strengthen the testing of asymptomatic infected persons starting April 1. Based on information released by the Health Commission of Hubei Province, Table 1 shows the asymptomatic infection situation in Hubei Province from March 31 to the end of the study period (8). During the investigation period from April 23 to 25, there were still about 20 new asymptomatic infected people every day, and the total number under medical observation was about 550. Therefore, the mental state of local people was still affected by this stress factor.

**Table 1:** Situation of asymptomatic COVID-19 infection in Hubei Province by the end of the investigation.

**Date**	**31 Mar**	**1 Apr**	**2 Apr**	**3 Apr**	**4 Apr**	**5 Apr**	**6 Apr**	**7 Apr**	**8 Apr**
Newly increased	47	37	51	38	23	35	18	30	24
Under medical observation	982	721	742	729	715	705	690	670	674
**Date**	**9 Apr**	**10 Apr**	**11 Apr**	**12 Apr**	**13 Apr**	**14 Apr**	**15 Apr**	**16 Apr**	**17 Apr**
Newly increased	18	17	20	17	22	32	37	32	33
Under medical observation	675	673	647	621	610	619	620	610	589
**Date**	**18 Apr**	**19 Apr**	**20 Apr**	**21 Apr**	**22 Apr**	**23 Apr**	**24 Apr**	**25 Apr**	
Newly increased	22	24	20	28	21	22	20	19	
Under medical observation	576	549	544	548	546	544	553	572	

A total of 2,340 participants were invited to fill out an electronic questionnaire on their cell phones using their real names. All participants were frontline service employees of Hubei Huangshang Co., Ltd., the largest supermarket chain in Huanggang. Specific jobs included sales, transportation, store management, and logistics support, while senior management staff was excluded. All participants were in contact with the flow of customers. Thus, we assumed they all had similar degrees of COVID-19 exposure risk and no occupational stratification was set. During the worst period of the epidemic, the company experienced the closure of some stores and it staggered work schedules to allow employees to reduce work hours. After the epidemic situation had eased, the work hours increased. This study was carried out during the process of staff resuming work. After excluding 31 incomplete questionnaires, 2,309 valid questionnaires were obtained with a response rate of 98.7%.

The study was approved by the institutional review board of the Ethics Committee of Beijing University of Chinese Medicine (2020BZHYLL0113), in accordance with the principles in the Declaration of Helsinki. All participants provided written informed consent.

### Questionnaire Design

Part A collected the demographic information of the participants, including gender, age, education, marital status, and whether they had school-aged children. Lifestyle information included smoking, drinking alcohol, drinking tea, and athletic activity. Health information included serious diseases and a history of allergies.

Part B assessed the knowledge and practice of COVID-19 prevention measures and concerns about returning to work. The question “Do you know the basic prevention measures for COVID-19?” was measured on a scale of 1 (know very much) to 5 (know nothing). The question “What do you know about basic preventive measures against COVID-19?” gave participants 10 options to see how well they understood each preventive measure. The question “Do you practice these prevention measures in your daily life?” was measured on a scale of 1 (absolutely practice them”) to 5 (practice none at all). Regarding whether participants had concerns about resuming work, they were asked to respond “yes” or “no.” If they answered “yes,” they could choose the possible reasons.

Part C investigated attitudes about using TCM to prevent COVID-19. TCM played an important role in treating COVID-19 in China. Confidence in the efficacy of TCM against COVID-19 was measured on a scale of 1 (very confident) to 5 (not confident at all). Participants were also asked if they needed preventive TCM products, and they could respond through a scale which ranged from 1 (need very much) to 4 (do not need at all). In addition, they were asked about their preference from among three forms of TCM dosage. Lastly, participants were asked about concerns they may have about TCM prevention and any related information they wanted to receive.

### Statistical Analysis

Data were analyzed using Stata for Windows, version 15 (College Station, Texas, USA). The significance level was set at 5%. Descriptive statistics were used to summarize the variables. Frequency and percentage were used for categorical variables, while median and quartile were used for the continuous variable “age,” which does not fit the normal contribution. Inferential statistics, including Wilcoxon rank-sum test and Chi-square (χ^2^) tests, were used to compare differences between groups with and without concerns about resuming work. Pearson's χ^2^, likelihood ratio χ^2^, Cramér's V, Goodman and Kruskal's γ, and Kendall's τb were used to measure the association between knowledge and practice of COVID-19 prevention (9–12). Logistic regression was used to analyze the factors affecting concerns about resuming work.

## Results

### Characteristics of Participants, Prevalence, and Characteristic Associations of Concerns About Resuming Work

Table 2 shows the demographic, lifestyle, and health characteristics of the participants and their associations with concerns about resuming work. In total, 1,421 participants (61.5%) expressed concerns about resuming work. The results showed that gender significantly affected concerns about resuming work, differences according to gender (*p* < 0.001), with women (64.2%) more likely to experience concerns about resuming work than men (47.4%). The participants with school-aged children were more concerned than those without school-aged children (*p* < 0.001). Besides, those who were pregnant or preparing for pregnancy were more concerned than those who were not (*p* = 0.005). There were significant relationships between lifestyle characteristics and concerns about resuming work (*p* < 0.01). The smokers were less likely to be concerned about resuming work than non-smokers (41.1 vs. 62.4%, respectively); the same result was revealed for those who drank compared with those who did not drink tea (53.7 vs. 62.5%, respectively). For health characteristics, those with a history of allergies were more concerned about resuming work than those without such a history (68.1 vs. 60.8%, respectively; *p* = 0.032). Table 3 shows the possible reasons for concerns about resuming work. The original intention was that participants who chose “yes,” i.e., concerned about resuming work, would indicate their reasons, but a mistake in the questionnaire caused those who chose “no” to give their reasons instead. Thus, the number of respondents for this question was 1,421, but we still present the result here for reference. Asymptomatic infection (85.01%) and employees gathering in the workplace (78.96%) were the most common reasons for concern.

**Table 2:** Characteristics and associations with concerns about resuming work.

**Variables**	**All participants (*N* = 2,309)**	**Concerns about resuming work** ** (** ***N*** **=** **1,421)**	
	**N(%)**	**N(%)**	***p*-value**
Age, median (IQR)	44 (37, 49)	44 (38, 48)	0.66
Gender			<0.001^**^
Male	361 (15.6%)	171 (47.4%)	
Female	1,948 (84.4%)	1,250 (64.2%)	
Marital status			0.41
Unmarried	127 (5.5%)	69 (54.3%)	
Married	2,054 (89.0%)	1,274 (62.0%)	
Divorced	76 (3.3%)	49 (64.5%)	
Widowed	25 (1.1%)	15 (60%)	
Remarried	27 (1.2%)	14 (51.9%)	
Education			0.087
Primary school and below	52 (2.2%)	29 (55.8%)	
Middle school	1,044 (45.2%)	669 (64.1%)	
High school	919 (39.8%)	556 (60.5%)	
College	227 (9.8%)	133 (58.6%)	
University	67 (2.9%)	34 (50.7%)	
Pregnancy preparation (included male)/			0.005^**^
pregnancy/ lactation			
No	2,228 (96.5%)	1,359 (61.0%)	
Yes	81 (3.5%)	62 (76.5%)	
Have school-aged children			<0.001^**^
No	1,085 (47.0%)	617 (56.9%)	
Yes	1,224 (53.0%)	804 (65.7%)	
Serious disease			0.47
No	2,278 (98.6%)	1,400 (61.5%)	
Yes	32 (1.4%)	21 (65.6%)	
Smoke			<0.001^**^
No	2,219 (96.1%)	1,384 (62.4%)	
Yes	90 (3.9%)	37 (41.1%)	
Drink alcohol			0.3
No	2,268 (98.2%)	1,399 (61.7%)	
Yes	41 (1.8%)	22 (53.7%)	
Drink tea			0.008^**^
No	2,065 (89.4%)	1,290 (62.5%)	
Yes	244 (10.6%)	131 (53.7%)	
Sports			0.09
Frequently (≥ 4 times/week)	442 (19.1%)	267 (60.4%)	
Sometimes (2~3 times/week)	1,010 (43.7%)	602 (59.6%)	
Seldom (≤ once/week)	857 (37.1%)	552 (64.4%)	
Allergy history			0.032^*^
No	2,083 (90.2%)	1,267 (60.8%)	
Yes	226 (9.8%)	154 (68.1%)	

** *p < 0.01*,

* *p < 0.05. Wilcoxon rank-sum test was used to analyze the non-normally distributed continuous variable “age.” Pearson's chi-squared tests were used to analyze other categorical variables*.

**Table 3:** Specific reasons for concerns about resuming work (*N* = 1,421).

	***N***	**%**
Asymptomatic infection is difficult to detect but is still infectious	1,208	85.01
A large number of employees gather in the workplace	1,122	78.96
Contact with too many people during commute	881	62.00
Global epidemic makes imported cases inevitable	776	54.61
Workplace/commuter transport cannot be thoroughly disinfected	731	51.44
Drugs for prevention and treatment are not being developed fast enough	533	37.51
Others^*^	6	0.42

* *Included some did not wear/incorrectly wore masks (two participants); income decreased (two participants); supermarket has large customer flow; inadequate protective measures*.

### Knowledge and Practice of COVID-19 Prevention

**Figure 2:**
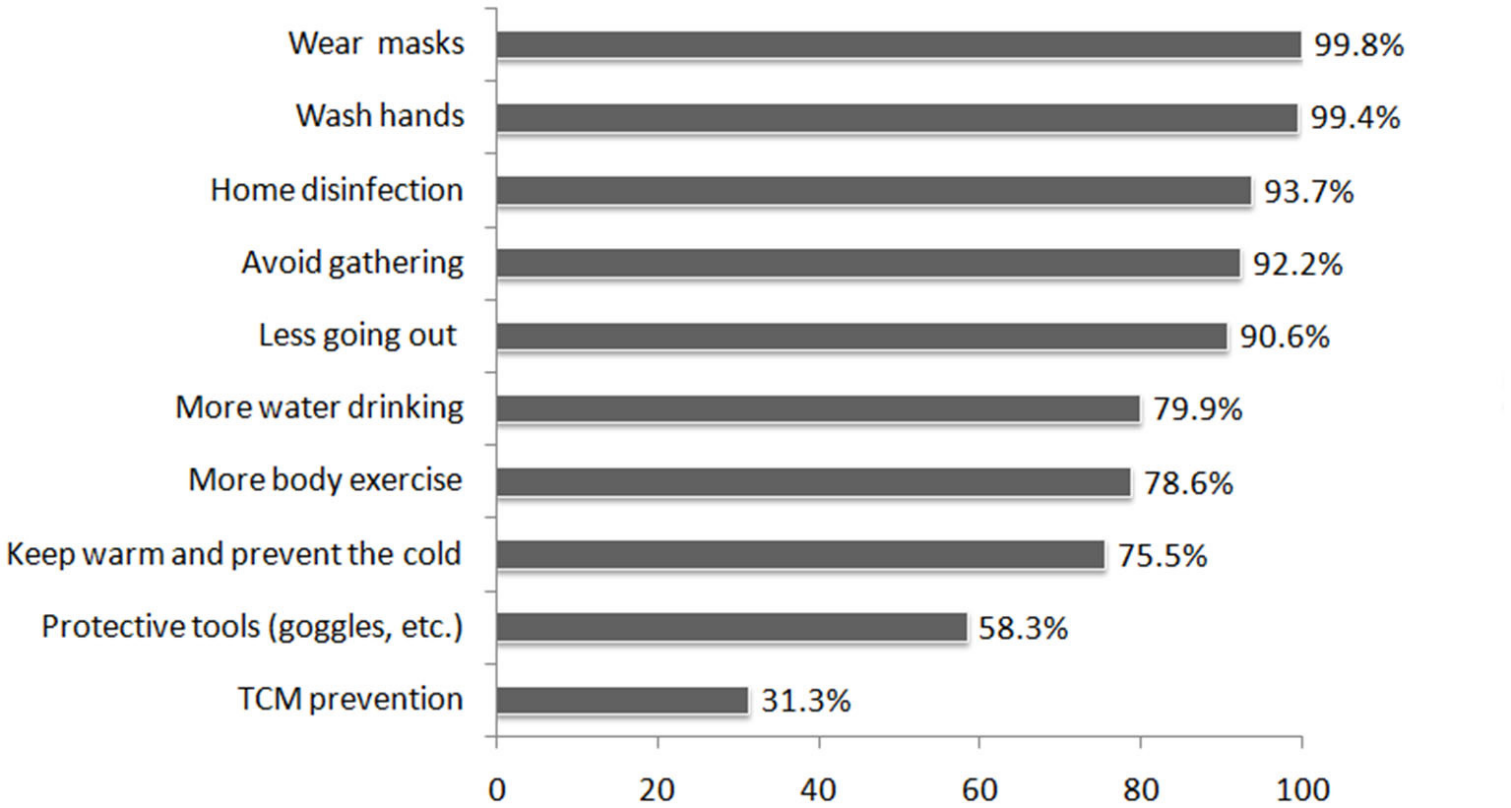
Knowledge rates of COVID-19 prevention measures.

A five-point Likert scale questionnaire (better to worse) was utilized for subjective evaluations of the knowledge and practice of preventive measures of the participants. Fifteen participants chose 4 (know little) or 5 (know nothing) in the question about knowledge of preventive measures, while 16 chose 4 (practice little) or 5 (practice nothing) in the question about practicing preventive measures. Since the amount was too small, levels 4 and 5 were combined into level 3, thus forming a total of three ordinal categories which were renamed as “Good,” “Moderate,” and “Worse.” Table 4 shows a cross-table of the knowledge and practice of the participants of COVID-19 prevention measures. The overall situation was relatively good, where 52.53% assessed their knowledge as moderate and 33.91% as good, and 57.82% assessed their practice as good and 39.24% as moderate. The Pearson's and likelihood ratio χ2 tests for the independence of the rows and columns showed a relationship between knowledge and practice of COVID-19 prevention (both *p* < 0.001). Cramér's V (from 0 to 1), Goodman and Kruskal's γ, and Kendall's τb (both go from −1 to 1) are measures of association between two nominal variables, where 1 indicates a strong association (13); V = 0.245, γ = 0.5319, and τb = 0.3073 indicated a statistically significant positive association. The row and column percentage also indicated that, regarding COVID-19 prevention, the better the knowledge, the better the practice (Table 4). Figure 2 shows the knowledge rate of COVID-19 prevention measures.

**Table 4:** Cross-table of knowledge and practice of COVID-19 prevention measures (*N* = 2,309).

		**Practice**			
		**Good**	**Moderate**	**Worse**	**Total**
**Knowledge**
Good	N	616	159	8	783
	row%	78.67	20.31	1.02	100
	column%	46.14	17.55	11.76	33.91
Moderate	N	608	577	28	1,213
	row%	50.12	47.57	2.31	100
	column %	45.54	63.69	41.18	52.53
Worse	N	111	170	32	313
	row%	35.46	54.31	10.22	100
	column%	8.31	18.76	47.06	13.56
Total	N	1,335	906	68	2,309
	row%	57.82	39.24	2.94	100
	column%	100	100	100	100

*Pearson's χ^2^ = 277.2792, p = 0.000; likelihood ratio χ^2^ = 269.6255, p = 0.000; Cramér's V = 0.2450; Goodman and Kruskal's γ = 0.5319, ASE = 0.028; Kendall's τb = 0.3073, ASE = 0.018*.

### Concerns About Resuming Work Associated With Knowledge and Practice of COVID-19 Prevention Measures and the Influencing Factors

Table 5 shows that concerns about resuming work differed with regard to the knowledge and practice of COVID-19 prevention measures of participants (both *p* < 0.001). The proportions of good knowledge and good practice in the group concerned about resuming work were lower than those in the group without concerns (30.3 vs. 39.8% and 53.9 vs. 64.1%, respectively). The proportions of worse knowledge and worse practice in the concerned group were higher than those in the unconcerned group (15.3 vs. 10.7% and 3.9 vs. 1.5%, respectively). Including the statistically significant variables that emerged in differences in concerns about resuming work, a multivariate ordinal logistic regression model was built to analyze the influencing factors (Table 6). Female staff was 1.78 times more likely to be concerned about resuming work than male staff (OR = 1.775, 95% CI, 1.367–2.304). Those with school-aged children were 1.4 times more likely to have concerns than those without (OR = 1.418, 95% CI, 1.194–1.684); similar results were revealed for those who were pregnant or preparing for pregnancy (OR = 1.88, 95% CI, 1.105–3.197). Knowledge and practice of preventive measures were also influencing factors. With worse knowledge and practice, there were more concerns about resuming work. Those with moderate and worse knowledge were 1.3 times (OR = 1.307, 95% CI, 1.078–1.586) and 1.58 times (OR = 1.572, 95% CI, 1.17–2.114), respectively, more likely to be concerned than those with good knowledge. Participants with moderate and worse practices were 1.3 times (OR = 1.345, 95% CI, 1.115–1.623) and 2.66 times (OR = 2.659, 95% CI, 1.413–5.005), respectively, more likely to feel concerned than those with good practices.

**Table 5:** Association between concerns about resuming work and knowledge and practice of COVID-19 prevention measures.

	**Concerned about resuming work**		
	**No (*N* = 888)**	**Yes (*N* = 1,421)**	***p*-value**
**Knowledge of prevention**
Good	353 (39.8%)	430 (30.3%)	<0.001^**^
Moderate	440 (49.5%)	773 (54.4%)	
Worse	95 (10.7%)	218 (15.3%)	
**Practice of prevention**
Good	569 (64.1%)	766 (53.9%)	<0.001^**^
Moderate	306 (34.5%)	600 (42.2%)	
Worse	13 (1.5%)	55 (3.9%)	

** *p < 0.01*.

**Table 6:** Characteristics and COVID-19 prevention knowledge and practice correlated with concerns about resuming work.

	**OR**	**95% CI**	***p*-value**
**Gender**			
Male	1		
Female	1.775	1.367–2.304	0.000^**^
**Pregnancy preparation/pregnancy/lactation**			
No	1		
Yes	1.88	1.105–3.197	0.020^*^
**Have school-aged children**			
No	1		
Yes	1.418	1.194–1.684	0.000^**^
**Smoke**			
No	1		
Yes	0.718	0.439–1.175	0.188
**Drinks tea**			
No	1		
Yes	0.833	0.629–1.103	0.203
**Allergy history**			
No	1		
Yes	1.285	0.953–1.734	0.100
**Knowledge of COVID-19 prevention**			
Good	1		
Moderate	1.307	1.078–1.586	0.007^**^
Worse	1.572	1.17–2.114	0.003^**^
**Practice of COVID-19 prevention**			
Good	1		
Moderate	1.345	1.115–1.623	0.002^**^
Worse	2.659	1.413–5.005	0.002^**^

** *p < 0.01*,

* *p < 0.05*.

### Attitudes and Needs Regarding TCM COVID-19 Prevention

Table 7 shows the results for all six questions related to TCM and COVID-19. The results showed that 73.4 and 65.8% of participants had positive attitudes (extremely confident and confident, respectively), regarding treating and preventing COVID-19 using TCM. Compared to treatment, confidence in TCM for prevention was slightly lower. A total of 43.8% of participants (highest proportion) had answered “Yes, more need” with regard to needing TCM products to prevent COVID-19, while 26% selected “No need” (second-highest proportion). Regarding the preferred form of TCM product, 71.5% selected oral granules. Toxicity (68.4%) and adverse reaction (53.7%) were the most common concerns regarding TCM products. Suitable population (70.8%) and clinical efficacy (70.7%) were the most common types of information participants wanted.

**Table 7:** Attitudes and need regarding TCM COVID-19 prevention (*N* = 2,309).

	***N***	**%**
**Do you have confidence in TCM treatment for COVID-19?**		
Extremely confident	582	25.2
Confident	1,112	48.2
Moderate	572	24.8
A little bit	38	1.6
None	5	0.2
**Do you have confidence in TCM prevention of COVID-19?**		
Extremely confident	361	17.7
Confident	980	48.1
Moderate	649	31.8
A little bit	287	14.1
None	32	1.6
**If preventive TCM products are developed by an authority, will you have**		
**a need for them?**		
No	601	26.0
Yes, limited need	524	22.7
Yes, more need	1,011	43.8
Yes, extreme need	173	7.5
**Which dosage form do you prefer for preventive TCM products?**		
Oral granules	1,650	71.5
Wearing sachet	403	17.5
Oral spray for external use	241	10.4
Others^*^	15	0.6
**What are your concerns about using TCM to prevent COVID-19?**		
No concerns	469	20.3
**(Among respondents with concerns** ***N*** **=** **1,840)**		
Toxicity of drugs	1,258	68.4
Chinese medicine has a bitter taste	548	29.8
Inconvenient to take the medicine	635	34.5
Cause adverse reaction	988	53.7
Difficult to adhere to medication	605	32.9
Others^**^	9	0.5
**What information would you like to know about using TCM to**		
**prevent COVID-19**		
Main mechanism of preventive products	1,426	61.8
Research and development	785	34
Suitable population	1,635	70.8
Clinical efficacy	1,632	70.7
Adverse reactions	1557	67.4
Others^***^	4	0.2

* *answered “herbal tea”*;

** *answered “preventive efficacy,” “high price,” “risk for pregnant or nursing women,” and “stimulate stomach or other organs”*;

*** *answered “whether the government announces it”*.

## Discussion

This study was conducted after the peak of the COVID-19 epidemic in China during a general trend of resuming work and production, although there were still asymptomatic infections. The study focused on the knowledge and practice of supermarket staff of preventive measures and concerns about returning to work. To our knowledge, this is the first study to find that 61.5% of supermarket staff were concerned about resuming work, indicating that this is a problem that needs attention and guidance. Concerns about asymptomatic infection (85.01%) and employees gathering in the workplace (78.96%) were major reasons, which are consistent with the actual situation of asymptomatic infection (8). Many studies measured mental status using anxiety, depression, and insomnia scales in the early and peak of the COVID-19 outbreak and identified the psychological effects on different populations (14–18). However, our study was conducted after the peak of the outbreak when work began to resume, and the prevalence of those psychological symptoms would thus be lower wherein the value of analysis decreased. Therefore, it was more meaningful to directly investigate concerns about resuming work. In addition, this is the first survey of its kind to assess supermarket staff. They were less likely to face the pressure of unemployment and were all exposed to the flow of people without the choice of working online. We found that about 85% of staff were women and most participants had middle to high school level education. This is consistent with the employment situation of supermarkets. Women are more attentive and patient and are suitable for sales, cashier, and inventory work, accounting for most of the work in supermarkets. While male staff are more inclined to manual labor and often work as drivers or security guards, and the number of jobs is less than those which women could acquire.

Being a female, having school-aged children, and being pregnant or preparing for pregnancy were risk factors for concerns about resuming work. Some COVID-19-related studies have reported a higher prevalence of mental health problems among women than men. Women showed a higher fear of COVID-19 infection (19), a higher likelihood of anxiety, depression, and/or PTSD symptoms (20), and higher PTSS in the domains of re-experiencing, negative mood, and hyperarousal (21). Female employees were less willing to return to the physical workplace during COVID-19 (22). In terms of pregnancy events, conditions such as extreme stress, emergency and conflict situations, and natural disasters could increase the risk of perinatal mental health morbidity. The financial uncertainties created by the prolonged pandemic are likely to further escalate psychological burdens and worsen the mental well-being of pregnant women and new mothers (23, 24). In our study, concerns about resuming work may have been more related to concerns regarding the well-being of the unborn or newborn child. Similarly, those who had a school-aged child were more likely to feel concerned about resuming work. Third-grade junior high school students in Huanggang first returned to school on May 26, which means school-aged children were still at home during the study period (25). Possible reasons for concerns about resuming work could have been separation anxiety and the stress created by the lack of time to secure childcare or education (26, 27). Demographic risk factors suggested greater understanding and support for female staff, especially those who were pregnant or preparing for pregnancy and those who had school-age children in their families. Psychological counseling, extra allowances, and flexible policies for resuming work can be offered as support for these supermarket staff working in high-risk areas.

In this study, we also found that better knowledge and practice of COVID-19 prevention measures reduced concerns about resuming work. One study investigated PTSD symptoms, stress, anxiety, depression, and insomnia among people returning to work after the COVID-19 quarantine and found that various preventative measures, e.g., hygiene, wearing masks, good workplace sanitization, reduced adverse psychiatric symptoms (28). Providing accurate information and informing the public that the outbreak can be controlled through protective behaviors were found to be associated with a lower prevalence of depression and anxiety (29). Our study found that a positive correlation between the knowledge and practice of preventive measures. A good understanding of preventive measures is the premise for promoting practice. This is consistent with a previous study that found, using multiple logistic regression, that COVID-19 knowledge was significantly associated with preventive practices (30). Among preventative measures, wearing masks and washing hands ranked highest for knowledge. These findings further highlighted the need for health education intervention (31, 32) and emphasized that the promotion of the knowledge and practice of preventive measures should not be relaxed.

Regarding how to do a better job of prevention, we casted our sights on TCM. TCM has played an important role in fighting COVID-19 in China (33, 34). Literature mining and analysis found that TCM has the potential to prevent and treat COVID-19. The network pharmacological studies demonstrated that TCM played roles of anti-virus, anti-inflammation, and immunoregulation *via* multiple components acting on multiple targets and multiple pathways. Clinical trials also confirmed the beneficial effects of TCM on the treatment of COVID-19 (35). TCM has entered the official new coronary pneumonia diagnosis and treatment scheme of China from the third edition and has been updated until the eighth edition. Most of the provinces also issued TCM prevention and treatment schemes that meet the characteristics of local patients (36, 37). A community-based randomized controlled trial (RCT) during COVID-19 found that the use of herbal medicine therapy could significantly reduce the risks of the common cold among community-dwelling residents (38). A single-arm study showed a good preventive effect of TCM decoction on close contacts of patients with COVID-19, which recommended to use Chinese medicine in such public health incidents in time and in order to effectively reduce the conversion rate of close contacts to confirmed patients (39). Even after the development of a vaccine has been completed and the injection rate has increased, taking into account the penetration rate and medical burden, TCM is still a very worthy prevention method to choose. Accordingly, 73.4% of respondents had a positive attitude about TCM treatment for COVID-19. In terms of prevention, 65.8% were confident about TCM. Although many areas of China have implemented preventive TCM programs, e.g., drug prevention, sachet prevention, and daily life adjustment, it still lags behind TCM treatment in terms of popularization and publicity. The knowledge rate of preventive measures also indicated that preventive TCM only accounted for 31.3%. Regarding the need for preventive TCM products, 74% of respondents indicated such a need and 51.3% a relatively large need. This suggested that preventive TCM products could have good market prospects. In terms of dosage form, 71.5% preferred oral granules; this is related to the perception that TCM involves drinking decoction. Finally, the concerns and information need of the participants regarding preventive TCM focus on safety and efficacy.

This study has some limitations. First, there was a mistake in the survey, as described in the results. Second, as one of the risk factors, “having school-aged children” was originally designed to investigate concerns about children returning to school. That was removed because of imperfect variable settings, which likely caused some confusion about why respondents were being asked about “school-aged” children but not preschoolers. Third, concerns about resuming work were closely related to the outbreak situation, and a cross-sectional survey can only capture a short period of time. Better control of the epidemic can not only relieve the concerns of the people about resuming work but also promote hope for economic recovery. Therefore, we cannot ignore that the most important factor influencing concerns about resuming work was the status of the epidemic.

## Conclusion

The findings of this study shed light on the need to be aware of the concerns of supermarket staff when they resume work. The experience from Huanggang showed that better knowledge and practice of preventive measures could protect against concerns about resuming work. Meanwhile, being female, having school-aged children, and being pregnant or preparing for pregnancy were risk factors. Both education and strengthened implementation of COVID-19 prevention measures are recommended to relieve concerns and ensure an orderly and efficient return to work. It is hoped that the study can contribute to providing a reference for the resumption of work in other countries or regions. Finally, this study is the first research to find positive attitudes about, and demand for, TCM products for COVID-19 prevention. The use of TCM could help to diversify prevention methods for COVID-19.

## Data Availability Statement

The raw data supporting the conclusions of this article will be made available by the authors, without undue reservation.

## Ethics Statement

The study was approved by the institutional review board of the Ethics Committee of Beijing University of Chinese Medicine (2020BZHYLL0113), in accordance with the principles in the Declaration of Helsinki. All participants provided written informed consent.

## Author Contributions

LLi, YM, and JW: study design, data collection, data analysis, and manuscript writing. YZe, HX, JH, ZL, SH, TL, JQ, YF, and WG: data collection. YZha, HL, and LLiu: data analysis guidance. YL, YZhe, and QW: study design and gave direction to the paper. All authors contributed to the article and approved the submitted version.

## Funding

This work was supported by the Key research and development project of the Ministry of Science and Technology (2020YFC0845200); Strategic Research and Consulting Project of Chinese Academy of Engineering(2021-XBZD-1).

## Conflict of Interest

The authors declare that the research was conducted in the absence of any commercial or financial relationships that could be construed as a potential conflict of interest.

## Publisher's Note

All claims expressed in this article are solely those of the authors and do not necessarily represent those of their affiliated organizations, or those of the publisher, the editors and the reviewers. Any product that may be evaluated in this article, or claim that may be made by its manufacturer, is not guaranteed or endorsed by the publisher.
