# Supplementary material for: Effect of Knowledge/Practice of COVID-19 Prevention Measures on Return-to-Work Concerns; Attitudes About the Efficacy of Traditional Chinese Medicine: Survey on Supermarket Staff in Huanggang, China
Source: Front Public Health. 2021 Sep 16;9:722604. doi: 10.3389/fpubh.2021.722604 (PMC8481610; doi:10.3389/fpubh.2021.722604)
Supplement: Supplementary file 1 [file Data_Sheet_1.PDF]

Questionnaire for Effect of Knowledge/Practice of COVID-19 Prevention Measures on Return-to-work Concerns; Attitudes about Traditional Chinese Medicine's Efficacy

Dear participant:

We are the research institution of this project, the National Institute of Traditional Chinese Medicine Constitution and Prevention of Diseases, Beijing University of Chinese Medicine. First of all, thank you very much for taking time out of your busy schedule to participate in our project. We will investigate your knowledge and practice on COVID-19 prevention and your attitude towards TCM prevention of COVID-19

Name：____________________

Phone number：____________________

ID number：____________________(get age information)

A. Demographic and lifestyle information

A1. Your gender is ______【ratio】

Male	01	Female	02

A2. What is your marital status 【ratio】

Unmarried	01	Widowed	04
Married	02	Remarried	05
Divorced	03		

A3. What is your highest educational background?【ratio】

Below primary school	01	College	05
Primary school	02	University	06
Middle school	03	Graduate	07
High school	04	Others_________	08

A4. Do you have a pregnancy plan in the last six months or are you pregnant or lactating?【**ratio**】

Yes	01
No	02

A5. Do you have a serious disease?【**ratio**】

No	01
Yes,____________________	02

A6. Do you have following living habits?【**multiple**】

Smoking	01
Drink alcohol	02
Drink tea	03

A7. How often do you exercise 30 minutes or more per week?【**ratio**】

Frequently (≥4times/week)	01
Sometimes(2~3times/week)	02
Seldom(≤once/week)	03

A8. Do you have a allergic history?【**ratio**】

No	01
Yes,____________________	02

B . Knowledge & Practice of COVID19 Prevention/ Return-to-work concern

B1. Are you familiar with the basic preventive measures for COVID-19?【**ratio**】

01	Know very much	Don't know much	04
02	Know well	Know nothing	05
03	General		

B2. What do you know about basic preventive measures against COVID-19?【**multiple**】

Wear masks	01	More water drinking	06
Wash hands	02	Keep warm and prevent the cold	07
Less going out	03	Protective tools(goggles,etc)	08
Home disinfection	04	TCM preventions	09
Avoid gathering	05	More body exercise	10

B3. What do you know about basic preventive measures against COVID-19?【**ratio**】

Absolutely practice them	01	Practice little	04
Practice more	02	Practice none at all	05
General	03		

B4. Do you have any concerns about the resumption of work?【**ratio**】

Yes	01	➔【**Go ahead**】
No	02	➔【 **To B6**】

B5. What are your specific reasons for concerns about resuming work ?【**multiple**】

A large number of employees gather in the workplace	01
Workplace/commuter transport cannot be thoroughly disinfected	02
Contact with too many people during commute	03
Global epidemic makes imported cases inevitable	04
Asymptomatic infection is difficult to detect but is still infectious	05
Drugs for prevention and treatment are not being developed fast enough	06
Others________________	07

B6.Do you have any school-aged child ?【**ratio**】

Yes	01	➔ 【Go ahead】
No	02	➔ 【 To C1】

B7. Do you have any concerns about children returning to school ? 【**ratio**】

Yes	01	➔ 【Go ahead】
No	02	➔ 【 To C1】

B8. What are your specific reasons for concerns about returning to school ? 【**multiple**】

Students' self-control ability is poor ,cannot keep good personal hygiene	01
A large number of students gathering bring higher risk	02
School area/commuter transport cannot be thoroughly disinfected	03
Contact with too many people during commute	04
Global epidemic makes imported cases inevitable	05
Asymptomatic infection is difficult to detect but is still infectious	06
The development of prevention and treatment drugs has not kept pace	07
Others_________________	08

C．Attitude about TCM prevention on COVID-19

C1. Do you have confidence in TCM treatment for COVID-19? 【**ratio**】

Extremely confident	01	A little bit	04
Confident	02	None	05
Moderate	03		

C2. Do you have confidence in TCM prevention of COVID-19? 【**ratio**】

Extremely confident	01	A little bit	04

Confident	02	None	05
Moderate	03		

C3.If preventive TCM products are developed by an authority, will you have a need for them?【**ratio**】

No need	Yes, limited need	Yes, more need	Yes, extreme need
1	2	3	4

C4. Which dosage form do you prefer for preventive TCM products?【**ratio**】

Oral granules	01	Oral spray for external use	03
Wearing sachet	02	Others________	04

C5. What are your concerns about using TCM to prevent COVID-19?【**Multiple**】

No concerns **[ratio]**	01
Toxicity of drugs	02
Chinese medicine has a bitter taste	03
Inconvenient to take the medicine	04
Cause adverse reaction	05
Difficult to adhere to medication	06
Others _________________	07

C6. What information would you like to know about using TCM to prevent COVID-19【**multiple**】

Main mechanism of preventive products	01
Research and development	02

Suitable population	03
Clinical efficacy	04
Adverse reactions	05
Others__________________	06

This is the end of the questionnaire, thank you very much for your support to our work, thank you!
